# MiR-1301-3p Inhibits Epithelial-Mesenchymal Transition via Targeting RhoA in Pancreatic Cancer

**DOI:** 10.1155/2022/5514715

**Published:** 2022-02-26

**Authors:** Xinxue Zhang, Zhangyong Ren, Junming Xu, Qing Chen, Jun Ma, Zhe Liu, Jiantao Kou, Xin Zhao, Ren Lang, Qiang He

**Affiliations:** Department of Hepatobiliary Surgery, Beijing Chao-Yang Hospital Affiliated to Capital Medical University, Beijing, China

## Abstract

Micro(mi)RNAs play an essential role in the epithelial-mesenchymal transition (EMT) process in human cancers. This study aimed to uncover the regulatory mechanism of miR-1301-3p on EMT in pancreatic cancer (PC). The miRNA profilings from Gene Expression Omnibus data sets (GSE31568, GSE41372, and GSE32688) demonstrated the downregulation of miR-1301-3p in PC tissues, which was validated with 72 paired PC tissue samples through qRT-PCR detection. The low level of miR-1301-3p was associated with a poor prognosis for PC patients from the PC cohort of The Cancer Genome Atlas and the validation cohort. Gene Ontology analyses indicated that the target genes of miR-1301-3p were involved in cell cycle and adherent junction regulation. *In vitro* assays revealed that miR-1301-3p suppressed the proliferation and migration abilities of PC cells. Western blotting and luciferase reporter assays suggested that miR-1301-3p inhibited RhoA expression by targeting its 3′-untranslated region; RhoA upregulated N-cadherin and vimentin levels; however, it downregulated the E-cadherin level. In conclusion, our study showed that miR-1301-3p could serve as a prognostic biomarker for PC and suppress PC cell malignancy by targeting the RhoA-induced EMT process.

## 1. Introduction

Pancreatic cancer (PC) is the fourth leading cause of cancer-related deaths worldwide with a 5-year overall survival (OS) of 5% [1]. Due to the lack of specific symptoms and biomarkers, 50% of patients were diagnosed with PC in the advanced stage and lost the opportunity for radical surgery [2]. Therefore, it is crucial to elucidate the signature driver molecules in PC tumorigenesis and progression. Micro(mi)RNA, a small noncoding RNA, can degrade the mRNA by binding to the 3′-untranslated region (3′UTR) of the target gene. Accumulating studies have revealed that miRNAs may regulate cancer-associated biological processes such as cell proliferation [3], differentiation [4], apoptosis [5], and epithelial-mesenchymal transition (EMT) [6].

We previously identified the clinically relevant miRNAs through conjoint analyses with multiple miRNA expression profiling data and found that miR-1301-3p was downregulated in PC tissues and the low level of miR-1301-3p was associated with poor OS for PC patients [7, 8]. Recent studies suggest that miR-1301-3p exhibits tumor-suppressive activity in esophageal squamous cell carcinoma [9], papillary thyroid carcinoma [10], and osteosarcoma [11] by interacting with noncoding RNAs. On the contrary, miR-1301-3p is upregulated in gastric cancer tissues and promotes cancer cell proliferation via targeting SIRT1 [12]. These results indicated possible dual regulatory roles of miR-1301-3p in various cancers; however, the effect of miR-1301-3p on PC is unclear and imperative to be discovered.

The purpose of this study was to validate the clinical significance of miR-1301-3p in PC and illustrate its function and potential signal pathway in PC cells.

## 2. Materials and Methods

### 2.1. The Differential and Survival Analyses for miR-1301-3p

Firstly, we extracted miR-1301-3p expression data from three PC-miRNA expression profilings (GSE31568, GSE41372, and GSE32688) of the Gene Expression Omnibus (GEO) database. Next, we compared the miR-1301-3p level between PC tissues and normal tissues within the three data sets. To verify the prognostic importance, the clinical data and miR-1301-3p expression value were also obtained through the PC cohort of The Cancer Genome Atlas (TCGA) database. According to the miR-1301-3p median value, we divided PC patients into high- and low-level groups and performed Kaplan–Meier survival analyses. The *P* value was calculated by the log-rank test.

### 2.2. Patients and Samples in the Validation Cohort

Between February 2018 and August 2020, 72 PC patients were enrolled in a validation cohort. These patients had not accepted radiotherapy or chemotherapy preoperatively, and the final diagnosis of PC was determined by pathological results. Surgically resected PC tissues and adjacent normal tissues were immediately stored in liquid nitrogen for two hours and then transferred into a −80°C refrigerator for storage. Postoperatively, these patients have followed an average of 12 months, ranging from two to 29 months. The Ethics Committee of Beijing Chao-Yang Hospital approved this study, and all patients signed the informed consent form.

### 2.3. Functional Annotation and Signaling Pathway Enrichment for miR-1301-3p

We first applied the miRWalk2.0 database to predict the binding genes of miR-1301-3p and then performed correlation analyses between the mRNA expression of these genes and miR-1301-3p values based on the PC cohort of TCGA. Finally, the genes negatively correlated with miR-1301-3p were regarded as the target genes of miR-1301-3p.

To understand the functions of these target genes, we performed Gene Ontology (GO) and Kyoto Encyclopedia of Genes and Genomes (KEGG) pathway enrichment analyses using the clusterProfiler *R* package [13]. A protein-protein interaction (PPI) network was constructed using the Search Tool for the Retrieval of Interacting Genes database and visualized with Cytoscape software. The correlation of each PPI relationship pair was represented by a combined score ranging from 0 (low) to 1 (high). In our study, an interaction >0.4 (moderate) was applied as the cut-off value. The Molecular Complex Detection plug-in in Cytoscape software was used to identify the hub genes among the PPI network. The screening conditions were set as degree cut-off = 2, K-Core = 2, and node score cut-off = 0.2. Besides, we verified the hub gene expression using the PC-mRNA data of GSE16515.

### 2.4. Quantitative Real-Time Polymerase Chain Reaction (qRT-PCR)

TRIzol (Invitrogen, USA) was used for total RNA extraction and qualified using the NanoDrop ND-1000 (Thermo Fisher, USA). Total RNA was converted to the first-strand cDNA according to the manufacturer's protocol (rtStar™ First-Strand cDNA Synthesis Kit; Arraystar Inc.). Specific primers for miR-1301-3p were designed by RiboBio (Guangzhou, China). The sequence of forward and reverse primers for miR-1301-3p was 5′- ACACTCCAGCTGGGTTGCAGCTGCCTGGGAGT-3′ and 5′-CTCAACTGGTGTCGTGGAGTCGGCAATTCAGTTGAGGAAGTCAC-3′. qRT-PCR was performed using Arraystar SYBR® Green Real-time qPCR Master Mix (Arraystar Inc.) according to the manufacturer's instructions. The relative expression of miR-1301-3p was calculated using the 2^−ΔΔCt^ method and normalized to *β*-actin expression levels.

### 2.5. Cell Culture and Transfection

**Figure 1:**
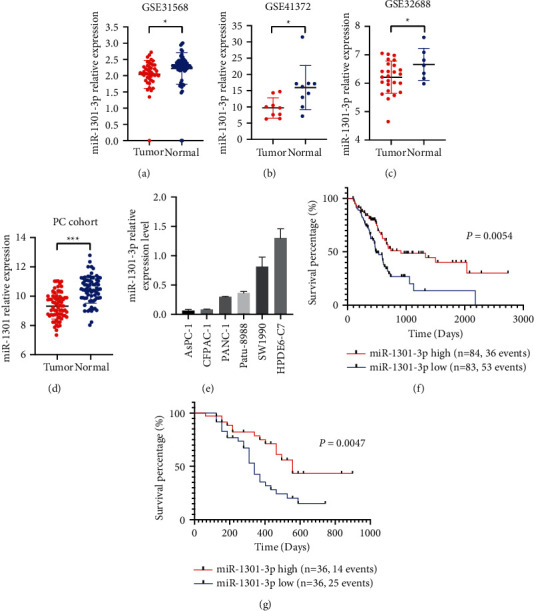
MiR-1301-3p was downregulated in PC tissues, and the low level of miR-1301-3p was associated with a poor prognosis for PC patients. (a–c) The miR-1301-3p expression level in PC tissues and the adjacent normal tissues in GSE31568, GSE41372, and GSE32688 data sets. (d) The miR-1301-3p expression level of the validation data set determined by qRT-PCR. (e) The relative miR-1301-3p expression level of normal pancreatic cell and PC cell lines. (f and g) The correlation between the miR-1301-3p level and the overall survival of PC patients from the PC cohort of TCGA and the validation cohort. ^*∗*^*P* < 0.05 and ^*∗∗∗*^*P* < 0.001.

Five PC cell lines and one pancreatic cell line were selected to test the expression of miR-1301-3p, including SW1990, AsPC-1, CFPAC-1, PANC-1, Patu-8988, and HPDE6-C7. These cells were purchased from the American Type Culture Collection (Manassas, VA, USA). We cultured these cells with DMEM (Biological Industries) containing penicillin/streptomycin and 10% fetal bovine serum (FBS) at 37°C with 5% CO_2_. Since miR-1301-3p was relatively highly expressed in SW1990 and PANC-1 cells (Figure 1(e)), we selected these two cell lines to conduct further experiments.

MiR-1301-3p mimics, inhibitor, and negative control (NC) were designed and synthesized by RiboBio (Guangzhou, China). Small interference RNA against RhoA (knockdown group, KD) was designed and synthesized by GenePharma (Shanghai, China). The lentiviral vectors encoding RhoA (overexpression group, OE) were constructed by GeneChem (Shanghai, China). In brief, miR-1301-3p mimics and inhibitor (100 nM) were transfected into PANC-1 and SW1990 cells using the Lipofectamine 3000 reagent (Invitrogen, USA) following the manufacturer's instruction.

### 2.6. Cell Counting Kit-8 (CCK-8) Assays

According to the manufacturer's instructions, we performed CCK-8 assays (Sigma Aldrich) to examine the proliferation ability of PC cells. Approximately 2 × 10^3^ cells were added to each well of the 96-well plate, and then the plate was cultured for 24 h at 37°C. Next, we added 50 *µ*l of the miR-1301-3p mimics, inhibitor, and NC to the wells of the 96-well plate. Then, the plate was placed in a 37°C incubator again for 24 h. At 0, 24, 48, 72, and 96 h, 10 *µ*l CCK-8 solution was added to each well. After 2 h optical density (OD), 450 nm values were measured using the enzyme-labeled instrument (Bio-Rad, United States). Cells were tested three times for each group.

### 2.7. Transwell Migration Assays

We conducted transwell migration assays with a chamber with 8 *μ*m pores (Corning, NY, USA). A suspension containing 1 × 10^4^ PANC-1 and SW1990 cells was prepared and suspended separately in serum-free DMEM with mitomycin-C (1 *μ*g/mL) and added into the upper chamber. After that, 500 *μ*l of 10% serum-containing DMEM was added into the lower chamber of the well and incubated 24 h at 37°C. After 24 h, PC cells in the upper chamber were removed. Four random fields were selected at 4× magnification for counting cell numbers. Each experiment was performed three times.

### 2.8. Wound Healing Assay

SW1990 and PANC-1 cells (1 × 10^5^) were incubated in six-well culture plates for 48 h until the cells were 80–90% confluent. Cells were maintained in 10% FBS containing DMEM media for 24 hours. PBS was used to wash away the nonadherent cells. A sterile 200-*µ*l pipet was used to make a scratch in the center of the cell monolayer. The monolayer was washed three times with PBS, and fresh media were added. After 0 h, 24 h, and 48 h, the wound width was measured at 2.5× magnification. Each assay was performed three times.

### 2.9. Protein Extraction and Western Blotting Assays

Total protein was extracted from PC cells after 72 h transfection, and the BCA protein assay kit (Beyotime, China) was used to measure protein concentration, followed by the manufacturer's instructions. Briefly, 12% SDS-PAGE was used for electrophoresis, and then the proteins were transferred to PVDF membranes. GAPDH, RhoA, E-cadherin, N-cadherin, and vimentin antibodies were used to analyze total protein. Specific primary rabbit anti-human antibodies (CST, 1 : 1000) were used to incubate the membranes at 4°C overnight. On the second day, the membranes were incubated with HRP-conjugated anti-rabbit IgG antibodies (1 : 2000) at room temperature for 1 h. An enhanced chemiluminescence detection system was used to visualize the bands. GAPDH was used as an internal control. Rabbit anti-GAPDH, RHOA, N-cadherin, E-cadherin, and vimentin antibodies (Cell Signaling Technology, Danvers, MA, USA) were used to analyze cell lysates.

### 2.10. Luciferase Reporter Assays

According to the starBase network tool, RhoA is a predicted target for miR-1301-3p. The binding site between miR-1301-3p and 3′UTR of RhoA was evaluated by using the pmirGLO dual-luciferase miRNA expression vector containing wild-type (WT) or mutant (MUT) 3′UTR of RhoA. The WT or MUT 3′UTR of RhoA and miR-1301-3p mimics were cotransfected into PANC-1 cells. After 48 h, the luciferase reporter assay system was used to examine the luciferase activity. Each experiment was performed in triplicate.

### 2.11. Immunohistochemistry Analyses

We applied immunohistochemistry assays to validate RhoA expression levels in PC tissues. In brief, we sectioned the paraffin-embedded tissue specimens and incubated them with anti-RhoA primary antibody (1 : 200; ZSGB-BIO, Beijing, China) overnight at 4°C. The slides were incubated with HRP-conjugated secondary antibody (goat anti-rabbit IgG; BOSTER, Hangzhou, China) for 1 h. To visualize the RhoA, 3,3′-diaminobenzidine substrate solution was used as the chromogen.

### 2.12. Statistical Analysis

R software, version 3.6.0, was used to perform statistical analyses. Continuous variables between the two groups were compared by a paired sample *t*-test. The data were presented as the mean ± standard deviation. Qualitative data were analyzed by the chi-square test. Fisher's exact test was employed to compare the categorical variables among groups. GraphPad Prism 8.0 (GraphPad Software, Inc., La Jolla, CA, USA) was applied to produce figures. *P* value < 0.05 was considered statistically significant.

## 3. Results

### 3.1. MiR-1301-3p Is Downregulated in PC Tissues and Its Low Level Is Correlated with a Poor Prognosis for PC Patients

Based on miRNA microarray data (GSE31568, GSE 41372, GSE32688), the miR-1301-3p was significantly downregulated in PC tissues, compared with normal tissues (Figures 1(a)–1(c)). In the validation cohort, the miR-1301-3p level was lower in PC tissues than in healthy tissues through qRT-PCR detection (Figure 1(d)). As shown in Figure 1(e), the miR-1301-3p level was relatively higher in the pancreatic cell than in PC cells. Furthermore, the miR-1301-3p low level was associated with poor OS in the PC cohort of TCGA and the validation cohort (Figures 1(f) and 1(g)). The univariate analyses showed that the miR-1301-3p low level was related to malignant pathological differentiation, tumor residual, and lymphatic metastasis in PC patients (Table 1).

### 3.2. MiR-1301-3p Inhibits the Proliferation and Migration Abilities of Pancreatic Cancer Cells

**Figure 2:**
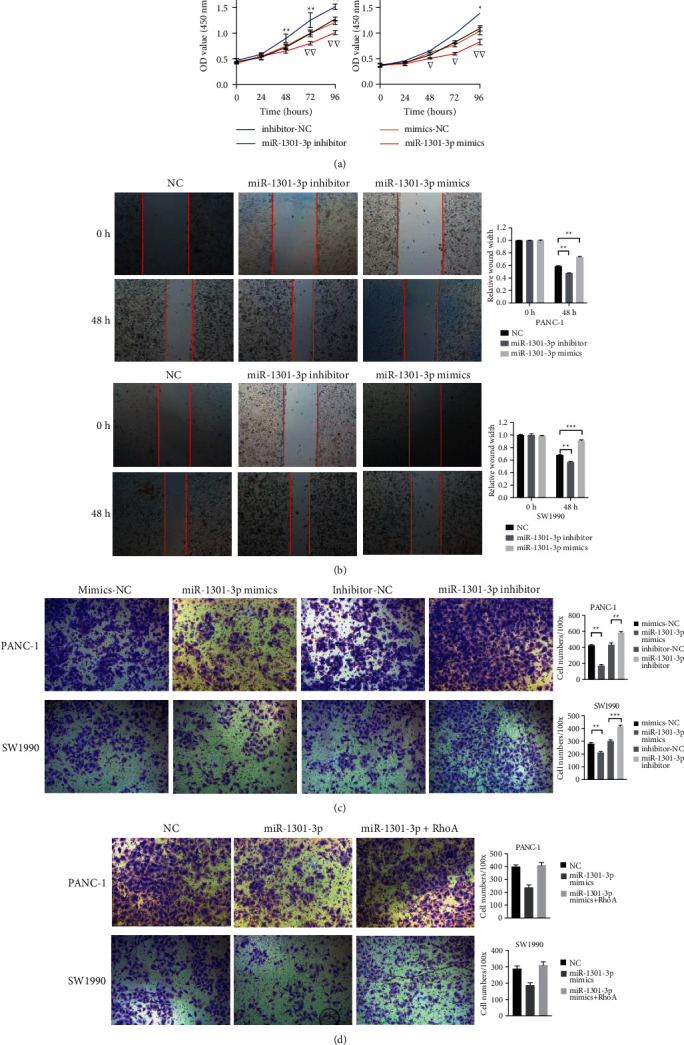
MiR-1301-3p inhibited the proliferation and migration ability of PC cells. (a) CCK-8 assays of PANC-1 and SW1990 cells transfected with miR-1301-3p mimics and inhibitor. (b) Wound healing assays of PANC-1 and SW1990 cells transfected with miR-1301-3p mimics and inhibitor. (c) Transwell migration assays of PANC-1 and SW1990 cells transfected with miR-1301-3p mimics and inhibitor. (d) Transwell migration assays of PANC-1 and SW1990 cells. Data were presented as the mean ± SD of three independent experiments. ^*∗*^*P* < 0.05, ^*∗∗*^*P* < 0.01, ^*∗∗∗*^*P* < 0.001, ▽*P* < 0.05, and ▽▽*P* < 0.01.

CCK-8 assays showed that PC cell proliferation was suppressed after miR-1301-3p mimics transfection (Figure 2(a)). Wound healing assays demonstrated that the migration ability of PC cells was lower in the miR-1301-3p mimics group than that in the NC group (Figure 2(b)). Similarly, transwell assays supported that miR-1301-3p mimics downregulated PC cell migration ability (Figure 2(c)). In addition, rescue experiments revealed that the inhibiting effect of miR-1301-3p on cell migration was reserved by RhoA overexpression (Figure 2(d)).

### 3.3. GO Annotation and KEGG Pathway Enrichment for MiR-1301-3p

**Figure 3:**
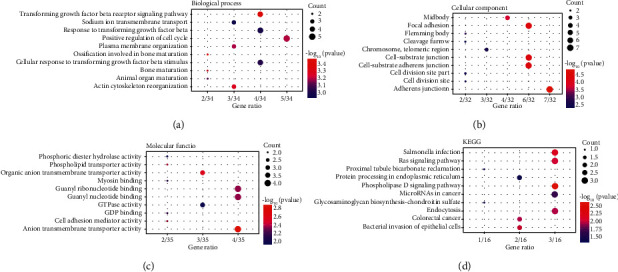
Functional annotation and pathway enrichment analysis of the target genes of miR-1301-3p. (a–c) Gene Ontology terms of biological process, cellular component, and molecular function. (d) Kyoto Encyclopedia of Gene and Genomes pathway enrichment for the miR-1301-3p target genes.

To uncover the potential functions of miR-1301-3p, we screened out 35 target genes of miR-1301-3p and performed bioinformatics analyses. The GO analyses showed that the target genes of miR-1301-3p were enriched in the positive regulation of cell cycle, TGF-*β* receptor signaling pathway, and cellular response to TGF-*β* stimulus in the biological process (Figure 3(a)). In terms of cellular components, the target genes were associated with adherens junctions, focal adhesion, and cell-substrate junctions (Figure 3(b)). In terms of molecular functions, the target genes were mostly enriched in anion transmembrane transporter activity and guanyl nucleotide binding (Figure 3(c)). The KEGG pathway analyses displayed that the target genes of miR-1301-3p were mainly enriched in the phospholipase D signaling pathway and Ras signaling pathway (Figure 3(d)). These results suggested that miR-1301-3p was probably associated with the regulation of cell migration and proliferation.

### 3.4. MiR-1301-3p Inhibits RhoA-Induced Epithelial-Mesenchymal Transition

**Figure 4:**
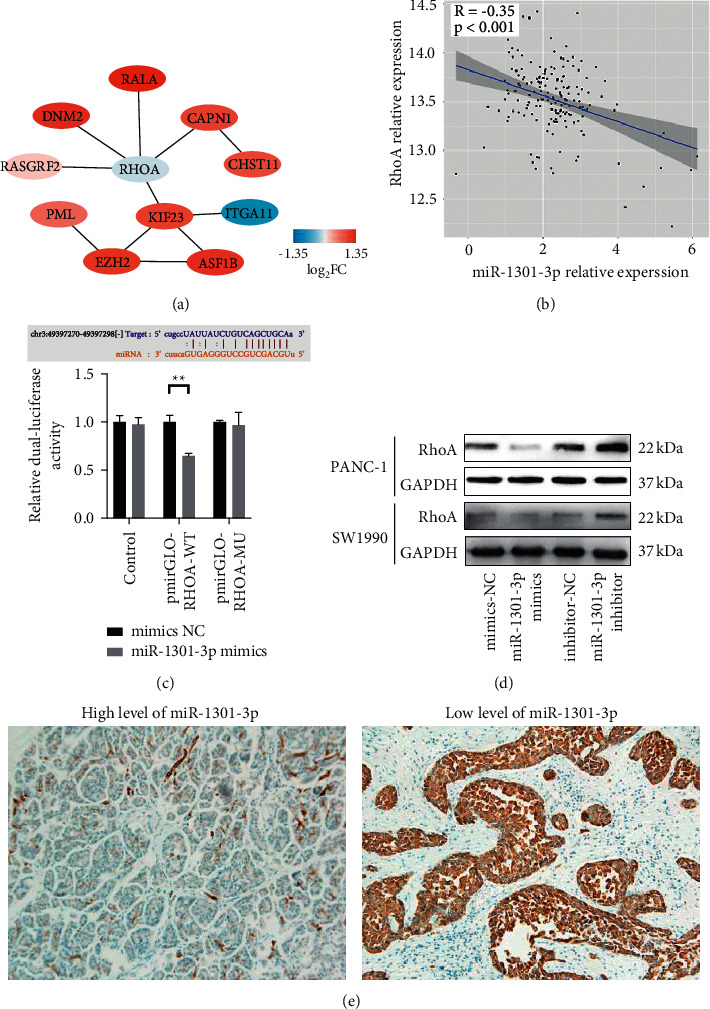
MiR-1301-3p suppressed RhoA expression in PC cells. (a) A protein-protein interaction network showed that RhoA acted as a hub gene among the miR-1301-3p target genes. (b) A negative correlation between miR-1301-3p and RhoA expression levels according to the PC cohort of TCGA. (c) Luciferase reporter assays demonstrated that miR-1301-3p was directly bound to the 3′UTR of RhoA in PANC-1 cells. (d) Western blotting assays showed that miR-1301-3p downregulated RhoA expression in PANC-1 and SW1990 cells. (e) The typical immunohistochemical staining of RhoA in miR-1301-3p-upregulated and miR-1301-3p-downregulated PC tissues.

We performed PPI analyses to screen out the pivotal gene in the 35 target genes of miR-1301-3p. After that, RhoA was identified as a hub gene in the PPI network (Figure 4(a)). Specially, RhoA is involved in the cellular junction and TGF-*β* receptor signaling pathway according to the GO and KEGG analyses, which were driving factors in tumor progression. Therefore, we selected RhoA to conduct the following validation experiments. As shown in Figure 4(b), miR-1301-3p negatively correlated with RhoA expression in the PC cohort of TCGA. Through the starBase network tool, the binding site was identified between miR-1301-3p and RhoA 3′UTR (Figure 4(c)). Moreover, luciferase reporter assays showed that miR-1301-3p mimics significantly downregulated the relative luciferase activity of RhoA-WT in PANC-1 cells (Figure 4(c)). Besides, WB assays showed that miR-1301-3p mimics decreased RhoA protein expression, while the miR-1301-3p inhibitor increased the RhoA level in PANC-1 and SW1990 cells (Figure 4(d)). Immunohistochemical examination indicated that RhoA staining was heavier in miR-1301-3p low-level PC tissue than in high-level PC tissue (Figure 4(e)).

**Figure 5:**
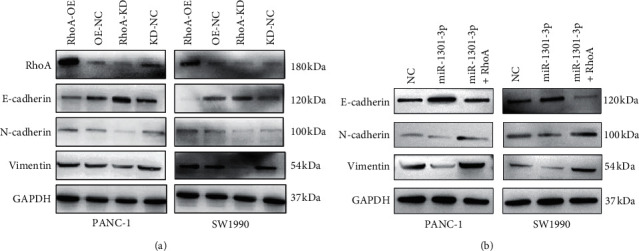
miR-1301-3p inhibited the epithelial-mesenchymal transition process via RhoA in PC cells. (a) The overexpression of RhoA upregulated N-cadherin and vimentin expression levels; however, it downregulated E-cadherin in PANC-1 and SW1990 cells. In contrast, RhoA knockdown downregulated N-cadherin and vimentin; however, it upregulated the E-cadherin level. (b) Western blotting assays showing the expression of E-cadherin, N-cadherin, and vimentin in pancreatic cancer cells.

Subsequently, we revealed that N-cadherin and vimentin expression levels were downregulated in the RhoA knockdown group than those in the NC group; in contrast, the E-cadherin level was upregulated in the RhoA knockdown group (Figure 5(a)). On the contrary, overexpression of RhoA increased N-cadherin and vimentin levels; however, it decreased the E-cadherin level in PANC-1 and SW1990 cells (Figure 5(a)). Further rescue experiments showed that RhoA overexpression could abolish the suppression of EMT process due to miR-1301-3p mimics (Figure 5(b)). Taken together, these results suggested that miR-1301-3p could inhibit RhoA-induced EMT in PC cells.

## 4. Discussion

Accumulated evidence has pointed out that miRNAs can contribute a crucial regulatory role in PC tumorigenesis and progression. Here, we revealed that miR-1301-3p was downregulated in PC tissues and its low level was related to the poor overall survival of PC patients. We also found that miR-1301-3p inhibited PC cell proliferation and migration abilities; mechanically, miR-1301-3p could suppress the RhoA-mediated EMT process in PC cells. Thus, our study provided a new molecular biomarker and a therapeutic target for PC treatment.

We reveal that the high level of miR-1301-3p is associated with good pathological differentiation, fewer infiltrating lymph nodes, and R0 resection in the current study. To our knowledge, we first discovered that miR-1301-3p may serve as a tumor suppressor in PC, combined with the results of *in vitro* assays. Generally, the growth of solid tumor relays on the tumor microenvironment that contains the complicated interactions between multiple stromal cells and the extracellular matrix. Thus, we failed to reveal that the overexpression of miR-1301-3p was related to smaller PC tumor, although *in vitro* assays suggested that miR-1301-3p suppressed PC cell proliferation.

GO analyses indicated that RhoA, a predicted target gene of miR-1301-3p, was involved in the terms of “TGF-*β* receptor signaling pathway,” “positive regulation of cell cycle,” “cellular response to TGF-*β* stimulus,” “focal adhesion,” and “myosin binding.” These GO terms suggested that miR-1301-3p possibly regulated cell proliferation and migration processes via RhoA. Furthermore, RhoA was involved in the “Ras signaling pathway” and “regulation of actin cytoskeleton” in KEGG enrichment analyses, which suggested that RhoA was linked to cell invasion ability. Therefore, we selected RhoA as a functional target gene of miR-1301-3p and conducted subsequent assays.

RhoA is a member of the Rho GTPase family, containing a GTP-bound active form and a GDP inactive part, which can promote actin cytoskeleton reorganization and regulate cell shape, attachment, and motility [14, 15]. The RhoA overexpression is associated with PC cell growth and metastasis. For example, KRas activation upregulated the eIF5A level, which promoted PC cells' motility and metastasis via Rho/ROCK [16]; cyclic AMP could decrease the RhoA level and inhibited PC cell migration and invasion [17]; and crizotinib, a MET antibody, could downregulate the RhoA level and suppress PC cell invasiveness [18].

A critical step of tumor metastasis is known as the EMT process, in which cancer cells lose their polarities and cellular connections and acquire migration ability [19, 20]. This process is characterized by loss of the cell adhesion protein, E-cadherin, and upregulation of N-cadherin and vimentin, representing mesenchymal phenotypes. It has been proven that EMT activators promote tumor development in multiple human cancers [21–24]. Activation of the RhoA/ROCK signaling pathway may upregulate the EMT process. Notably, RhoA was reported to facilitate the EMT process in gastric cancer and esophagus cancer [25, 26]; however, the association between EMT and RhoA is unclear in PC. In this study, we provided evidence that RhoA activation promoted the EMT process in PC cells. Interestingly, several miRNAs could also downregulate RhoA; for example, miR-154-3p and miR-487-3p specifically repressed RhoA expression and blocked thyroid cancer cell growth [27]; miR-101 downregulated the EMT process and breast cancer cell migration by reducing the RhoA level [28].

In summary, we revealed that miR-1301-3p could serve as a prognostic biomarker for PC. Overexpression of miR-1301-3p inhibits PC cell proliferation and migration. Mechanistically, miR-1301-3p suppresses the RhoA-induced EMT process, and thus, miR-1301-3p/RhoA could be a novel target for PC treatment.

## Figures and Tables

**Table 1:** Association between miR-1301-3p level and the clinicopathological parameters of pancreatic cancer patients in the study cohort.

Clinical parameters	miR-1301-3p		*P* value
	Low (*n ***=** 36)	High (*n ***=** 36)	
Age (years)	61	64	0.327
*Gender*			0.637
Male	21	18	
Female	15	18	
*Pathological differentiation*			**<0.001**
Moderate and high	18	33	
Poor	18	3	
Tumor size (cm)	4.0 ± 1.5	3.7 ± 2.1	0.608
*Resection*			**0.018**
R0	24	33	
R1 and R2	12	3	
Numbers of positive lymph node	5.0 ± 4.5	1.5 ± 1.8	**<0.001**
*Vascular invasion*			0.634
Negative	19	22	
Positive	17	14	
*TNM stage*			0.285
I and IIA	7	12	
IIB and IV	29	24	
*Primary tumor*			0.443
T1 and T2	23	27	
T3 and T4	13	9	
*Regional lymph nodes*			0.119
N0	7	14	
N1 and N2	29	22	
*Distant metastases*			0.107
Negative	30	35	
Positive	6	1	

## Data Availability

The clinical data used to support the findings of this study are available from the corresponding author upon request.
